# Prevalence of Common Respiratory Viral Infections and Identification of Adenovirus in Hospitalized Adults in Harbin, China 2014 to 2017

**DOI:** 10.3389/fmicb.2018.02919

**Published:** 2018-11-27

**Authors:** Yingchen Wang, Tuo Dong, Guiyun Qi, Lixin Qu, Wei Liang, Binbin Qi, Zhe Zhang, Lei Shang, Hong Gao, Xiqiao Du, Bing Lu, Yan Guo, Zhenwei Liu, Huisong Yu, Qi Cui, Xiaocen Wang, Ye Li, Weiyuan Guo, Zhangyi Qu

**Affiliations:** ^1^Department of Microbiology, Public Health College, Harbin Medical University, Harbin, China; ^2^Department of Clinical Laboratory, The Second Affiliated Hospital of Harbin Medical University, Harbin, China; ^3^Department of Ear Nose Throat, The Second Affiliated Hospital of Harbin Medical University, Harbin, China; ^4^Department of Natural Focus Disease Control, Institute of Environment-Associated Disease, Sino-Russia Joint Medical Research Center, Harbin Medical University, Harbin, China

**Keywords:** human adenovirus, respiratory viral infections, lower respiratory tract infections, adult patients, epidemiology

## Abstract

**Background:** Respiratory infections pose a great challenge in global health, and the prevalence of viral infection in adult patients has been poorly understood in northeast China. Harbin is one of the major cities in northeast China, and more than half of any given year in Harbin is occupied by winter. To reveal the viral etiology and seasonality in adult patients from Harbin, a 4-year consecutive survey was conducted in Harbin, China.

**Methods:** From January 2014 to December 2017, specimens were obtained from adult patients admitted to the Second Affiliated Hospital of Harbin Medical University with lower respiratory tract infections. Sputum samples were examined by direct immunofluorescence assays to detect seven common respiratory viruses, including influenza virus (type A and B), parainfluenza virus (type 1 to 3), respiratory syncytial virus and adenovirus. Adenovirus positive samples were seeded onto A549 cells to isolate viral strains. Phylogenetic analysis was conducted on the highly variable region of adenoviral hexon gene.

**Results:** A total of 1,300 hospitalized adult patients with lower respiratory tract infections were enrolled, in which 189 patients (14.5%) were detected as having at least one viral infection. The co-infection rate in this study was 25.9% (49/189). The dominant viral pathogen from 2014 to 2017 was parainfluenza virus, with a detection rate of 7.2%, followed by influenza virus, respiratory syncytial virus and adenovirus. Based on the climate seasons determined by daily average temperature, the highest overall viral detection rate was detected in spring (22.0%, 52/236), followed by winter (13.4%, 109/813), autumn (11.4%, 13/114) and summer (10.9%, 15/137). Adenovirus type 3 strains with slight variations were isolated from positive cases, which were closely related to the GB strain from the United States, as well as the Harbin04B strain isolated locally.

**Conclusion:** This study demonstrated that common respiratory viruses were partially responsible for hospitalized lower respiratory tract infections in adult patients from Harbin, China, with parainfluenza virus as the dominant viral pathogen. Climate seasons could be rational indicators for the seasonality analysis of airborne viral infections. Future surveillance on viral mutations would be necessary to reveal the evolutionary history of respiratory viruses.

## Introduction

Lower respiratory tract infections are a persistent public health problem, causing more than two million deaths per year worldwide, with a rate of 36 deaths per 100,000 population (GBD 2016 Causes of Death Collaborators, 2017). The morbidity and mortality of respiratory infections could be even worse in developing countries, including China. Viral infections played an important role in pediatric lower respiratory tract infections, and the corresponding common viral pathogens were influenza A and B virus (IAV and IBV), parainfluenza virus (PIV, type 1 to 3), respiratory syncytial virus (RSV) and human adenovirus (ADV) (Pavia, 2011). These seven viruses were also common in respiratory infections of the adult population in Shandong province, China (Liu et al., 2015). To the best of our knowledge, there were no commercial vaccines available for most of these viruses, except for influenza A and B viruses (Lambkin-Williams et al., 2018).

The etiology of respiratory infection in the adult population has been overlooked, at least in China, although there are plenty of reports on the epidemiology of respiratory viral infection in the pediatric population (Wang et al., 2016). China is a vast country with notable variations in climate characteristics among different regions. Due to the differences in socioeconomic, geographic or climate factors, the epidemiological features of viral infection in the respiratory tract show dramatic variation among different study populations (Sloan et al., 2011; Lu et al., 2013; He et al., 2014; Rehder et al., 2015).

Harbin is one of the major metropolises in northeast China, hosting more than eight million people. The winter in Harbin lasts for more than half a year, while the summer is short, resulting in the length of four seasons being unequal. Viral respiratory infections in the pediatric population from Harbin were reported by the authors’ team in 2009 (Zhang et al., 2009), in which the adult population was not included. A clear picture of the viral etiology in hospitalized adults with respiratory infections ought to be critical for both public health policy makers and clinical practitioners.

Phylogenetic history of several respiratory viruses isolated from China in recent years have been well established, including the influenza virus (Wang et al., 2018; Yang et al., 2018), the PIV (Pan et al., 2017) and the RSV (Liu et al., 2014; Zou et al., 2016). The information about adenoviral evolutionary history in China was limited to outbreak reports and novel strains identification (Lu et al., 2014; Li et al., 2018), leaving a gap for circulating stains among adult patients.

Human adenovirus is one of the common pathogens in respiratory infections and could be divided into seven species (Ismail et al., 2018). The most recent reported human adenovirus type is type 85 in species D, which was isolated from Japan (Hashimoto et al., 2018). Human adenovirus species B type 55 (HAdv B55) was reported to be responsible for adult pneumonia outbreaks in Beijing China during 2011 with the genome identity as 99.87%, comparing to the prototype strain of HAdv B55 in 2006 (Cheng et al., 2018). Adenoviral infections in children from Harbin were found to be ADV species B, as reported by the authors, while the viral type among adult patients is still poorly understood.

The adenoviral virion was composed of 252 capsomeres, containing 240 hexons and 12 pentons, while each penton was anchored by one fiber (or two fibers in several types) (Nemerow et al., 2012). The knob region in adenoviral fiber was responsible for cellular attachment through host receptors including CAR, CD46, DSG-1 and sialic acids (Baker et al., 2018; Lenman et al., 2018). The RGD loop of penton could be recognized by the host cell’s integrin (Veesler et al., 2014). The hexon protein is the major neutralizing antigen of adenoviruses (Madisch et al., 2005), while the hyper variable region (HVR) in hexon protein hosts its type specific epitopes reported by the authors’ team (Yuan et al., 2009). Experiments on chimeric adenoviral capsids showed that the replacement of HVRs in hexon could alter the viral serotypes (Qiu et al., 2012; Tian et al., 2018). The hexon specific CD4^+^ and CD8^+^ T cells have been proved to be an effective treatment for human adenoviral infections in clinical settings (Zandvliet et al., 2010; Feucht et al., 2015). Recent reports indicated that antibodies against fiber knob or penton base may also be involved in the neutralization of adenoviruses (Yu et al., 2013; Tomita et al., 2018). To the best of our knowledge, the hypervariable region of adenoviral hexon protein plays an essential role in viral type-specific immunity.

In this report, the prevalence of common viruses in the lower respiratory tract infection of hospitalized adult patients from Harbin, China was explored in hopes of revealing the clinical and pathogenic features of respiratory viruses. Adenoviral strains were isolated from positive cases to identify potential molecular variations within its hypervariable regions.

## Materials and Methods

### Specimens and Clinical Data Collection

From January 2014 to December 2017, sputum samples were collected from 1,300 adult patients hospitalized for lower respiratory tract infections in the Second Affiliated Hospital of Harbin Medical University. All patients enrolled in this study lived in urban and suburban areas of Harbin without travel histories within 3 months before admission and sampling. All patients were older than 18 years and suffering from at least one complaint, including fever with body temperature ≥ 38°C, cough, expectoration, hemoptysis, chest tightness (chest pain), or dyspnea. The sputum samples were expectorated spontaneously into sterile containers and delivered to the laboratory within 2 h, tested immediately or stored at -80°C prior to use.

Patients’ clinical data, including symptoms, history of illness, clinical diagnoses and laboratory test results were surveyed through the hospital’s health information system. This study had been approved by the ethical review committees of Harbin Medical University in accordance with the Declaration of Helsinki. Both informed and written consents were obtained from each participant who provided specimens.

### Detection of Respiratory Viruses

The presence of common respiratory viruses, including PIV type 1 to 3, influenza virus A and B virus (IAV and IBV), RSV, and human ADV was determined by the D^3^ Ultra^TM^ DFA Respiratory Virus Screening Kit (Diagnostic Hybrids, Inc., Athens, OH, United States).

### Cell Culture and Virus Isolation

Virus isolation for the ADV-positive specimens was performed using the A549 cell line (provided by the Cancer Hospital of Harbin Medical University) following the protocol described previously (Smith et al., 1986; Huang et al., 2013). In brief, Cells seeded with clinical samples were incubated at 37°C for 4 days. If there was no observed cytopathic effect, two additional blind passages of the culture were performed to avoid false negative results. The cultures with ADV-like cytopathic effects were passaged again to confirm viral presence.

### Preparation of Nucleic Acids

The viral DNA of human ADV was extracted from infected cells using the AxyPrep Multisource Genomic DNA Miniprep Kit (Axygen Biosciences) according to the manufacturer’s instructions and eluted in 100 μl elution buffer.

### PCR Amplification and Sequence Typing

The highly variable region in the adenoviral hexon gene was amplified using the following primer pairs: forward, 5′-CAATTCACTCGCTCCTA-3′ and reverse, 5′-GTGGAAAGGCACATAACG-3′. All primers were synthesized by Comate Bioscience Co., Ltd. PCR was performed with the Platinum PCR SuperMix (Invitrogen) following the manufacturer’s instructions. A total reaction volume of 50 μL contained 5 μL of 10 × PCR buffer (Takara Bio Premix Ex Taq^TM^), 200 nM of each primer, 200 nM of each dNTPS, 2 μL of each DNA sample, 0.25 μL Taq enzyme (Takara Bio Premix Ex Taq^TM^), and sterile water. The cycling conditions were 5 min at 95°C, followed by 35 cycles of 30 s at 95°C, 30 s at 55°C, and 30 s at 72°C, with a final 5-min extension at 72°C. DNA extracted from the A549 cell culture and sterile water were used as negative controls. The amplicons were bi-directionally sequenced using the Sanger sequencing method with an ABI PRISM 3130 genetic analyzer (Comate Bioscience Co., Ltd., Jilin, China).

### Air Temperature Data and Climate Seasons

The daily average air temperature from January 1st, 2014 to December 31st, 2017 in Harbin city was kindly provided by the Harbin Meteorological Observatory. Historical temperature datasets covering 1981–2010 in Harbin were accessible from the China Meteorological Data Service Center. Climate seasons were determined based on the smoothed daily average temperature curve according to the Chinese national standard No: QX/T 152-2012 (Administration, 2012). In brief, spring starts when the daily average temperature permanently rose above 10°C, while summer comes when it rose above 22°C permanently. The autumn starts when the daily temperature drops to 22°C permanently and the winter begins at 10°C. “Permanently” means the daily temperature has remained above or below the threshold for at least five consecutive days. The details can be found in the Supplementary Table S1.

### Statistical Analysis

Statistical analysis was conducted using the R language (version 3.4.2). The prevalence (detection percentage) of viruses was calculated by dividing the sum of positive cases by the number of cases in total. Chi-square tests or Fisher’s exact tests were selected for comparing the cross tables of categorical variables. The Wilcoxon rank-sum or Kruskal–Wallis tests were chosen for continuous variable comparisons as appropriate. Datasets were visualized by an Excel spreadsheet.

### Phylogenetic Analysis

The quality of sequencing data was checked manually via Chromas software version 2.4 (Technelysium Pty Ltd., South Brisbane, QLD, Australia), before contigs for each isolate were assembled using UGENE (Protsyuk et al., 2015) Software suite (version 1.9) guided by the published hexon gene sequence of human adenoviruses. Similar strains to the isolates in this report were found from the GenBank database via the BLAST search method (Boratyn et al., 2013). Fifty-eight nucleotide sequences of the hexon gene covering all seven species of human ADV were selected as the reference in this study. Before the tree building process, multiple sequences were aligned by MUSCLE (Edgar, 2004) software (version 3.6). The unrooted Neighbor-Joining tree was reconstructed using the kimura 2 parameters distance, whose robustness was tested by the bootstrap method with 1,000 replications implemented in MEGA (Kumar et al., 2018) software (version 10.0.1). The parsimony tree was also built on the same dataset.

## Results

### Demographic and Clinical Characteristics

In total, 1,300 hospitalized adult patients with respiratory infection were enrolled from January 2014 to December 2017 in Harbin city. Among these patients, 710 were male and 590 were female, as shown in Table 1. The age of the population in this study varied from 18 to 97 years old, while the median age was 61 with an interquartile range (IQR) from 51 to 71. These 1,300 patients were divided into three age groups: (1) young group (18–49 years old), 288 patients; (2) middle age group (50–69 years old), 652 patients; and (3) senior citizen group (>70 years old), 360 patients.

**Table 1:** Demographic and clinical characteristics summary of enrolled patients.

Characteristics	All patients n = 1300), no.(%)	Overall viral detection		*P*-value
			
		Positive, (*n* = 189), no.(%)	Negative, (*n* = 1111) no.(%)	
Male	710(54.6)	104(55.0)	606(54.5)	0.902
Age, median (IQR)	61(51-71)	63(54-69)	61(51-71)	0.730^a^
**Age groups in years**				0.104
18–49	288(22.2)	38(20.1)	250(22.5)	
50–69	652(50.2)	108(57.1)	544(49.0)	
≥70	360(27.7)	43(22.8)	317(28.5)	
**Surveillance year**				0.017
2014	299(23.0)	29(15.3)	270(24.3)	
2015	280(21.5)	52(27.5)	228(20.5)	
2016	327(25.2)	53(28)	274(24.7)	
2017	394(30.3)	55(29.1)	339(30.5)	
**Climate season**				0.003
Spring	236(18.2)	52(27.5)	184(16.6)	
Summer	137(10.5)	15(7.9)	122(11.0)	
Autumn	114(8.8)	13(6.9)	101(9.1)	
Winter	813(62.5)	109(57.7)	704(63.4)	
**Calendar season**^b^				0.002
Spring	481(37.0)	81(42.9)	400(36.0)	
Summer	203(15.6)	25(13.2)	178(16.0)	
Autumn	476(36.6)	52(27.5)	424(38.2)	
Winter	140(10.8)	31(16.4)	109(9.8)	
**Clinical diagnosis**				0.025
Pneumonia	556(42.8)	99(52.4)	457(41.1)	
Bronchitis	496(38.2)	69(36.5)	427(38.4)	
COPD	128(9.8)	11(5.8)	117(10.5)	
Asthma	20(1.5)	1(0.5)	19(1.7)	
Bronchiectasia	45(3.5)	5(2.6)	40(3.6)	
Other LRI	55(4.2)	4(2.1)	51(4.6)	
**Days of hospitalization, median (IQR)**	13(9-17)	12(9-19)	13(9-17)	0.306^a^
Chronic diseases^c^	583(44.8)	68(36.0)	515(46.4)	0.008
**Clinical signs**				
Fever	658(50.6)	91(48.1)	567(51.0)	0.463
Cough	628(48.3)	101(53.4)	527(47.4)	0.127
Expectoration	453(34.8)	70(37.0)	383(34.5)	0.494
Hemoptysis	49(3.8)	8(4.2)	41(3.7)	0.717
Chest tightness	606(46.6)	72(38.1)	534(48.1)	0.011
Dyspnea	343(26.4)	41(21.7)	302(27.2)	0.113


*^*a*^ Wilcoxon test. ^*b*^ Calendar seasons defined by Spring (February to April), Summer (May to July), Autumn (August to October) and Winter (November to January of the next year). ^*c*^ Chronic diseases refers to chronic respiratory disease, cancer, diabetes mellitus, chronic cardiac diseases, cerebrovascular disease, chronic kidney disease and other chronic disease lasting for more than 1 year.*

The hospital days for these patients varied from 1 to 242 days with a median of 13 days. Of all the patients, 556 were diagnosed with pneumonia, 496 with bronchitis, 128 with COPD (Chronic obstructive pulmonary disease), 45 with bronchiectasis, 20 with asthma and 51 with other infections. Some clinical signs were more frequent among cases with certain diagnoses. In all, 370 (66.5%) pneumonia cases complained of fever, while only 185 (37.3%) cases with bronchitis had fever (*P* < 0.001). Cough was the chief complaint of the bronchial infections (*P* < 0.001), including bronchitis (56.0%), COPD (55.5%) and asthma (60.0%). Out of 1,300 enrolled patients and 583 were diagnosed with chronic diseases, including chronic respiratory disease, cancer, diabetes mellitus, chronic cardiac diseases, cerebrovascular disease, chronic kidney disease, and other chronic diseases lasting more than 1 year.

### Detection Percentage of Seven Viruses

Among the 1,300 total patients, 189 (14.5%) cases were determined to be positive with at least one of seven viruses, including influenza virus (A and B), PIV (type 1, 2, and 3), RSV and ADV. One hundred and forty patients were infected with only one virus as the single infection, and 49 patients were co-infected with more than one virus. Double infections were observed in 43 cases, while triple infection was found in six cases. As shown in Table 2, the most frequent single infection was PIV (59 cases) and the major double infection was combined influenza and parainfluenza viruses (17 cases). The details were accessible from the Supplementary Table S2.

**Table 2:** Summary of detected viral infections (2014-2017).

Co-infection level	Flu	PIV	RSV	ADV	Cases
1-Single	+	-	-	-	43
	-	+	-	-	59
	-	-	+	-	32
	-	-	-	+	6
2-Double	+	+	-	-	17
	+	-	+	-	8
	+	-	-	+	3
	-	+	+	-	8
	-	+	-	+	5
	-	-	+	+	2
3-Triple	+	+	+	-	1
	+	-	+	+	2
	-	+	+	+	3
Total					189


*Flu (influenza virus, including type A and B), PIV (parainfluenza virus, including type 1 to 3), RSV (respiratory syncytial virus), and ADV (adenovirus).*

### Age and Gender Distribution

The median age of the viral positive group was 63 (IQR 54–69) and the negative group was 61 (IQR 51–71). The age difference between the viral infection positive and negative group was not statistically significant (*p* = 0.730). The viral detection rate varied from 16.6% (108/652) in the middle age group to 11.9% (43/360) in the senior citizen group, but the difference was not statistically significant (*p* = 0.104). There was no significant difference in the viral infections between genders (*p* = 0.902).

### Year and Seasonal Distribution

The virus detection rate among the study years varied significantly (*p* = 0.017) from 18.6% (52/280) in 2015 to 9.7% (29/299) in 2014, as shown in Table 1. The detection rate was not equal among calendar seasons (*p* = 0.002) or climate seasons (*p* = 0.003). Based on the climate seasons, the highest overall detection rate was in spring (22.0%, 52/236), followed by winter (13.4%, 109/813), autumn (11.4%, 13/114), and summer (10.9%, 15/137).

For individual virus positive samples, certain viruses were more frequently detected in spring than in other seasons, including influenza virus (8.9%, 21/236), PIV (9.7%, 23/236), and RSV (9.3%, 22/236), as shown in Table 3. ADV was more frequently detected in winter, with a detection rate of 1.8% (15/813). PIV was the dominant viral pathogen during the study period, accounting for 65.5% (19/29) of the viral infection cases in 2014, 40.3% (21/52) in 2015, 54.7% (29/53) in 2016, and 43.6% (24/55) in 2017. As shown in Table 3, viral co-infections were more not equally distributed among seasons, with the highest ratio in spring at 7.2% (17/236, *p* = 0.022).

**Table 3:** Respiratory virus detection percentages within clinical groups.

	Total cases (*n*)	Flu, *n*(%)	PIV, *n*(%)	RSV, *n*(%)	ADV, *n*(%)	Single infection, *n*(%)	Double infection, *n*(%)	Triple infection, *n*(%)	Positive cases, *n*(%)
Overall	1300	74(5.7)	93(7.2)	56(4.3)	21(1.6)	140(10.8)	43(3.3)	6(0.5)	189(14.5)
**Age in years**									
18-49	288	11(3.8)	16(5.6)	13(4.5)	7(2.4)	30(10.4)	7(2.4)	1(0.3)	38(13.2)
50-69	652	39(6.0)	56(8.6)	34(5.2)	11(1.7)	79(12.1)	26(4.0)	3(0.5)	108(16.6)
≥70	360	24(6.7)	21(5.8)	9(2.5)	3(0.8)	31(8.6)	10(2.8)	2(0.6)	43(11.9)
**Surveillance year**									
2014	299	12(4.0)	19(6.4)	9(3.0)	7(2.3)	14(4.7)	12(4.0)	3(1.0)	29(9.7)
2015	280	21(7.5)	21(7.5)	17(6.1)	5(1.8)	41(14.6)	10(3.6)	1(0.4)	52(18.6)
2016	327	19(5.8)	29(8.9)	12(3.7)	4(1.2)	42(12.8)	11(3.4)	0(0.0)	53(16.2)
2017	394	22(5.6)	24(6.1)	18(4.6)	5(1.3)	43(10.9)	10(2.5)	2(0.5)	55(14.0)
**Climate seasons**									
Spring	236	21(8.9)	23(9.7)	22(9.3)	4(1.7)	35(14.8)	16(6.8)	1(0.4)	52(22.0)
Summer	137	10(7.3)	5(3.6)	2(1.5)	1(0.7)	13(9.5)	1(0.7)	1(0.7)	15(10.9)
Autumn	114	6(5.3)	4(3.5)	5(4.4)	1(0.9)	10(8.8)	3(2.6)	0(0.0)	13(11.4)
Winter	813	37(4.6)	61(7.5)	27(3.3)	15(1.8)	82(10.1)	23(2.8)	4(0.5)	109(13.4)
**Clinical diagnoses**									
Pneumonia	556	35(6.3)	51(9.2)	31(5.6)	12(2.2)	73(13.1)	22(4.0)	4(0.7)	99(17.8)
Bronchitis	496	29(5.8)	30(6.0)	20(4.0)	9(1.8)	52(10.5)	15(3.0)	2(0.4)	69(13.9)
COPD	128	5(3.9)	9(7.0)	2(1.6)	0(0.0)	6(4.7)	5(3.9)	0(0.0)	11(8.6)
Asthma	20	0(0.0)	0(0.0)	1(5.0)	0(0.0)	1(5.0)	0(0.0)	0(0.0)	1(5.0)
Bronchiectasia	45	3(6.7)	2(4.4)	1(2.2)	0(0.0)	4(8.9)	1(2.2)	0(0.0)	5(11.1)
Other LRI	55	2(3.6)	1(1.8)	1(1.8)	0(0.0)	4(7.3)	0(0.0)	0(0.0)	4(7.3)


*Flu (influenza virus, including type A and B), PIV (parainfluenza virus, including type 1 to 3), RSV (respiratory syncytial virus), and ADV (adenovirus).*

### Daily Distribution With Air Temperature

**FIGURE 1:**
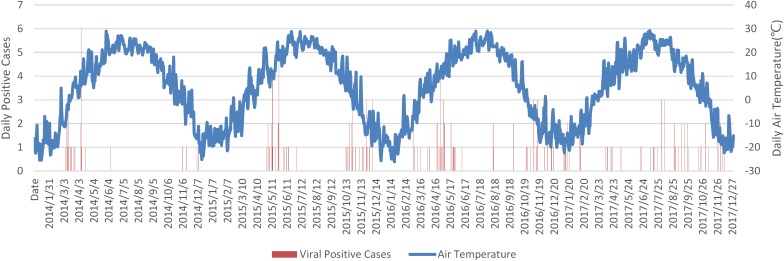
Daily overall viral positive cases against air temperature. Notes: Date string was formatted as YYYY/MM/DD.

Overall detected respiratory viral positive cases were plotted against daily average air temperature in Figure 1. The low temperature over days was a strong indicator for respiratory viral infections. Positive cases were rare in summer, which began late in June and ended in mid of August. One exception was found in the summer in 2017 which was related to a sudden drop of air temperature from 27.2°C on 18th July to 18.8°C on 27th July 2017.

### Viral Infection and Clinical Characteristics

The overall percentage of viral infection was significantly connected with clinical diagnoses (*p* = 0.025), as shown in Table 1. The highest detection rate was observed in the pneumonia group (17.8%, 99/556), followed by bronchitis (13.9%, 69/496), while the lowest detection rate was found in the asthma group (5%, 1/20). Chest tightness was the only symptom that showed statistically significant difference (*p* = 0.011) between the positive (38.1%, 72/189) and negative (48.1%, 534/1111) group of viral infections. The respiratory viral infection did not prolong hospital stay statistically (*p* = 0.306), although the median days in the hospital for the positive viral infection group was 12 (IQR 9–17), which was shorter than 13 in the negative group (IQR 9–17). Overall, the viral detection rate was relatively lower in the chronic diseases group (11.7% vs. 16.9%), with statistical significance (*p* = 0.008). Detection rates varied among different chronic diseases, but the difference was not significant (*p* = 0.467). The viral co-infection ratio among different diagnosed groups was not significant (*p* = 0.372).

### Isolation and Phylogenetic Analysis of Adenovirus

**FIGURE 2:**
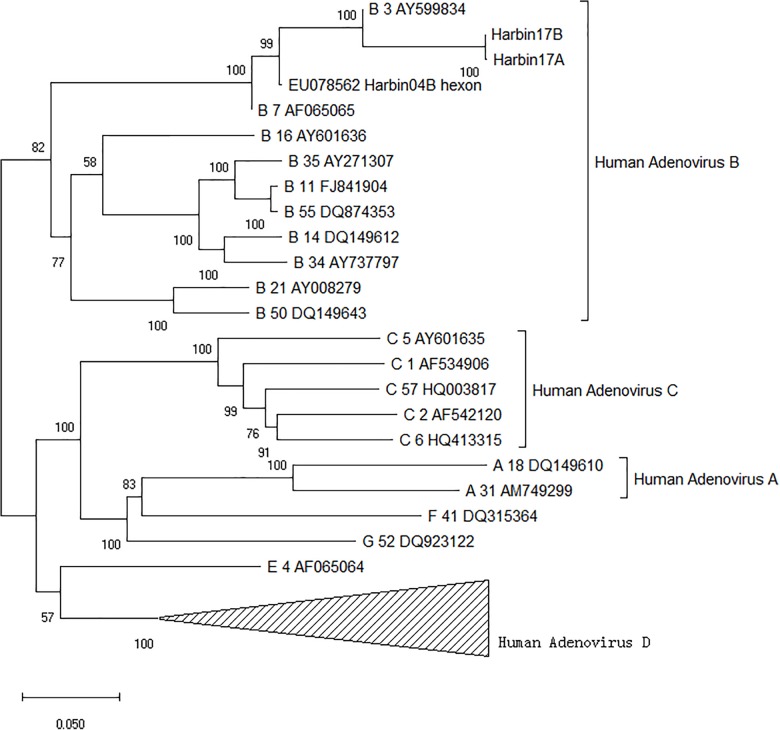
Phylogenetic tree of human adenoviruses based on the highly variable region in the hexon gene.

Two strains of human ADV were isolated from two patients diagnosed with pneumonia, respectively. The Harbin17B strain came from a male patient aged 69 whose samples were collected in December 2017. The other strain, named Harbin17A, was isolated from a male patient aged 24 whose samples were collected in January 2017. Based on the evolutionary tree reconstruction of the hexon gene sequencing data, both strains isolated in this study were identified as human ADV species B type 3. These two strains were nearly identical, with the closest evolutionary distance to the strain GB (GenBank: AY599834; ATCC: VR-3) from the United States as well as the Harbin04B strain from a previous local epidemic, as shown in Figure 2. The parsimony tree was similar in topology with neighbor-joining tree (figure not shown in the article). Multiple sequence alignments indicated that, compared with the GB strain, the Harbin17A and Harbin17B strain shared a variable site in the 482nd codon (GB numbering) of the hexon gene hosting an A to C mutation, resulting in threonine (T) to proline (P) mutation of the amino acid sequences. The Harbin04B strain had a threonine on the same site in the hexon protein encoded by the codon ACU, which was identical to the Harbin17A and Harbin17B strain.

## Discussion

Adult populations with respiratory infections bear heavy burdens of hospitalization due to severe respiratory illness, especially for senior citizens (Muller-Pebody et al., 2006). The constantly changing nature of viral pathogens has made the surveillance of these infections critical to public health authorities (Zou et al., 2016; Li et al., 2017). The key indicators for viral infections from this report and recent published surveys in China has been summarized in Table 4. The overall detection rate of viral infection among hospitalized adult patients in this report is 14.5%, which was consistent with the result of 16.8% in the age group above 14 years old by a national survey from 2009 to 2013 in China (Feng et al., 2014). The dominant viral pathogen throughout this study period was PIV, with a detection rate of 7.2%, followed by influenza virus. The national survey on hospitalized patients with respiratory infection in China from 2009 to 2013 showed that influenza virus had the highest detection rate of 8.0% in adult patients. The detection rate of respiratory viruses in the over 16 years age group was 24.2% in Shandong province of China (Liu et al., 2015) and was dominated by influenza virus (8.7%) from 2011 to 2013. From 2012 to 2015, the respiratory viral detection rate in hospitalized patients above 14 years old within Beijing and Shandong China was reported to be 38.9%, in which influenza virus had the highest frequency of 8.0% (Yu et al., 2018). Compared with recent surveys in Beijing, Shandong and other regions in China, this report showed that the PIV dominated epidemic seasons from 2014 to 2017 in adult patients in Harbin, which should be important for preparing future epidemic control strategies in public health.

**Table 4:** Summary of detected viral infections in adult patients among this study and recent reports.

Surveillance area	Surveillance period	Age in years	Total cases	Viral positive cases	Flu A and B (%)	PIV (%)	RSV (%)	ADV (%)	hBoV (%)	HMPV (%)	HcoV (%)	HRV (%)	Co-detection (%)	Reference
Harbin	2014-2017	≥18	1300	189(14.5)	74(5.7)	93(7.2)	56(4.3)	21(1.6)	-(-)	-(-)	-(-)	-(-)	43(3.31)	This report
Shandong	2011-2013	≥16	240	58(24.2)	21(8.7)	15(6.3)	5(2.1)	6(2.5)	-(-)	1(0.4)	12(5.0)	6(2.5)	6(2.5)	Liu et al., 2015
Beijing and Shandong	2012-2015	≥14	1431	556(38.9)	114(8.0)	84(5.9)	37(2.6)	78(5.5)	28(2.0)	47(3.3)	54(3.8)	114(8.0)	-(-)	Yu et al., 2018
24 Provinces in China	2009-2013	≥15	7732	1300(16.8)	625(8.1)	133(1.7)	125(1.6)	132(1.7)	26(0.3)	64(0.8)	107(1.4)	-(-)	88(1.1)	Feng et al., 2014


*Flu (influenza virus, including type A and B), PIV (parainfluenza virus, including type 1 to 3), RSV (respiratory syncytial virus), ADV (adenovirus), hBoV (Human bocavirus), HMPV (Human metapneumovirus), HCoV (Human coronavirus), HRV (Human Rhinoviruses), “-”, not reported.*

The viral infection rate within asthma subgroup of this report was 5%, and it was one patient with RSV among 20 asthma patients in total as shown in Table 3. Asthma is related to viral infection, while the detection rates varies among different pathogens. A recently published meta-analysis showed that the mean prevalence of RSV, influenza virus, PIV and ADV was 13.6%, 10%, 5.6%, and 3.8%, respectively (Zheng et al., 2018). The apparently low detection rate within asthma group in this report could be possibly caused by the sampling bias due to the limited subgroup size.

Air temperature was a major meteorological variable associated with respiratory viral infection (Chan et al., 2015). Harbin city is located at 46 degrees north latitude with an annual average temperature of 4.9 degrees Celsius. Climate seasons are not in equal length in Harbin, according to historical statistics from 1981 to 2010. The longest season in Harbin is winter, which starts on the 4th of October and lasts 201 days, followed by spring on the 23rd of April with 61 days. Starting on the 23rd of June, summers in Harbin are relatively short at 53 days, while autumn is 50 days and is the shortest season, starting on the 15th of August. In routine epidemiological analysis, 12 months in a year were usually divided into four seasons equally, which is not the case in Harbin and possibly compromises contagious disease seasonality prediction. Based on climate seasons defined by the daily average air temperature, influenza, parainfluenza and RSV detected in this report all presented significant seasonal peaks in spring, although similar phenomena were absent within the calendar seasons.

Sputum samples were acceptable both in viral culture and antigen detection in respiratory infections compared with nasopharyngeal swabs and aspirates (Covalciuc et al., 1999), although they were not frequently collected in recent surveys on lower respiratory tract infected adult patients from China. Detection of respiratory virus in sputum samples has been proven effective on a small scale, either by the immunofluorescence method (Honkinen et al., 2012) or the PCR method (Branche et al., 2014). This report provided a medium scale test on the validity or effectiveness of respiratory viral detection from sputum samples with 1,300 cases.

In this report, two nearly identical strains were isolated from pneumonia patients in Harbin, possessing high similarity with the local strain Harbin04B and the strain GB. The Harbin04B strain was isolated from a pediatric patient in Harbin (Zhang et al., 2009). ADV strain GB was named VR-3 in the ATCC database, which was isolated from an adult patient with common cold in Maryland, United States in 1953 (Huebner et al., 1954). The similarity of these strains across Asian and American continents through several decades may result from the globalization movement in modern China. It could also be explained by another hypothesis based on the evolution history of adenoviruses. On the geological scale, Homo sapiens inherit human adenoviruses from our ancestors within the Hominidae, resulting in an evolutionary rate of human ADV as low as 2.8E-8 substitution per site per year (Hoppe et al., 2015). Considering its 36 kbp genome, we would have to wait a millennium (or 992 years exactly) before a single site mutation within an adenoviral genome occurred naturally.

There were a few limitations to this study. First, the 4 years of investigation on a single city in this present report was relatively short and much more localized than other long-term or national surveillance, which may lead to a bias of the viral etiology. Second, the enrolled population in this study consisted of adult patients only, while the samples from pediatric patients were not collected simultaneously. The comparison of the viral prevalence status between adult and pediatric populations in this study was difficult. Third, the bacterial culture results of the clinical samples were not included in this report, which prevents us from drawing a comprehensive picture on the pathogens associated with lower respiratory tract infections.

## Conclusion

In this report, 14.5% of lower respiratory tract infections in hospitalized adults from Harbin were found to be related with common respiratory viruses. The PIV dominated viral infection from 2014 to 2017 with detection rates of 7.2%. Climate seasons based on daily temperature records were found to be reliable in seasonality analysis. ADV type 3 strains with slight variations were isolated from positive cases. Further surveillance would be important to continuously monitoring the viral etiology in adult patients both local and abroad.

## Author Contributions

ZQ, YW, and TD designed this study and prepared the manuscript. GQ, YG, and WG collected the specimens. YW, TD, and YG conducted the epidemiological investigation. TD, LQ, WL, BQ, ZZ, LS, HG, XD, BL, ZL, HY, QC, XW, and YL performed the laboratory experiments. YW and TD analyze the data and made the figures and tables. All authors reviewed the manuscript.

## Conflict of Interest Statement

The authors declare that the research was conducted in the absence of any commercial or financial relationships that could be construed as a potential conflict of interest.
