# Supplementary material for: Prevalence of Common Respiratory Viral Infections and Identification of Adenovirus in Hospitalized Adults in Harbin, China 2014 to 2017
Source: Front Microbiol. 2018 Nov 27;9:2919. doi: 10.3389/fmicb.2018.02919 (PMC6277751; doi:10.3389/fmicb.2018.02919)
Supplement: Supplementary file 1 [file Data_Sheet_1.PDF]

Supplementary Table 1 Daily average air temperature among climate and calendar seasons in Harbin 2014-2017

Year	Seasons	Climate Seasons		Daily Average Temperature(C°)			Calendar Seasons		Daily Average Temperature(C°)		
		Start Date	Duration (in days)	Median(IQR)	Lowest	Highest	Start Date	Duration (in days)	Median(IQR)	Lowest	Highest
2014	Spring	22-Apr	61	17.7(14.1 ~ 21.1)	5.1	29.0	1-Feb	89	8.0(1.9 ~ 14.4)	-11.4	29.0
	Summer	22-Jun	53	23.1(21.2 ~ 25.0)	18.9	27.1	1-May	92	22.3(20.8 ~ 24.4)	18.9	28.0
	Autumn	14-Aug	48	18.0(14.9 ~ 21.5)	5.1	24.2	1-Aug	92	6.5(-0.8 ~ 14.3)	-10.3	22.1
	Winter	1-Oct	203	-6.4(-16.9 ~ 4.2)	-25.4	18.0	1-Nov	92	-17.7(-20.4 ~ -14.4)	-25.4	5.0
2015	Spring	25-Apr	51	18.3(12.0 ~ 19.6)	5.4	24.4	1-Feb	89	8.4(1.4 ~ 12.9)	-15.1	24.4
	Summer	15-Jun	66	24.4(22.8 ~ 25.5)	17.9	28.8	1-May	92	23.1(20.9 ~ 25.0)	14.2	28.8
	Autumn	20-Aug	53	17.0(11.7 ~ 20.3)	6.9	22.4	1-Aug	92	7.7(-2.3 ~ 14.9)	-14.8	22.4
	Winter	12-Oct	195	-7.7(-14.3 ~ 2.1)	-25.7	18.0	1-Nov	92	-14.3(-17.3 ~ -11.2)	-25.7	0.9
2016	Spring	21-Apr	74	18.0(13.9 ~ 20.4)	7.7	25.5	1-Feb	90	8.8(3.0 ~ 13.9)	-9.7	25.2
	Summer	4-Jul	53	23.9(22.6 ~ 26.4)	18.7	29.0	1-May	92	22.6(20.0 ~ 25.2)	16.0	29.0
	Autumn	26-Aug	40	18.0(14.8 ~ 19.4)	5.5	22.1	1-Aug	92	4.5(-6.7 ~ 16.1)	-18.0	22.1
	Winter	5-Oct	199	-7.8(-15.4 ~ 2.1)	-26.1	16.1	1-Nov	92	-15.2(-18.3 ~ -12.3)	-26.1	1.8
2017	Spring	29-Apr	57	17.6(13.2 ~ 21.3)	4.7	21.3	1-Feb	89	7.8(1.6 ~ 14.9)	-14.2	26.0
	Summer	25-Jun	61	24.8(23.2 ~ 26.2)	18.8	29.2	1-May	92	23.2(19.9 ~ 25.5)	11.9	29.2
	Autumn	25-Aug	47	15.3(11.6 ~ 17.0)	4.8	21.3	1-Aug	92	6(-0.7 ~ 14.2)	-17.6	21.0
	Winter	11-Oct	200	-7.8(-15.4 ~ 3.0)	-22.4	16.5	1-Nov	92	-15.5(-18.2 ~ -12.8)	-22.4	0.9

Notes: Climate seasons determined in accordance with the Chinese national standard No: QX/T 152-2012.

Supplementary Table 2 Co-infection summary based on 7 common respiratory viruses

Co-Infection Level	FluA	FluB	PIV1	PIV2	PIV3	RSV	ADV	Cases
1-Single	+	-	-	-	-	-	-	21
	-	+	-	-	-	-	-	22
	-	-	+	-	-	-	-	11
	-	-	-	+	-	-	-	28
	-	-	-	-	+	-	-	14
	-	-	-	-	-	+	-	32
	-	-	-	-	-	-	+	6
2-Double	+	-	+	-	-	-	-	2
	+	-	-	+	-	-	-	3
	+	-	-	-	+	-	-	4
	+	-	-	-	-	+	-	3
	+	-	-	-	-	-	+	1
	-	+	+	-	-	-	-	3
	-	+	-	+	-	-	-	1
	-	+	-	-	+	-	-	2
	-	+	-	-	-	+	-	4
	-	+	-	-	-	-	+	1
	-	-	+	+	-	-	-	2
	-	-	+	-	+	-	-	2
	-	-	+	-	-	+	-	3
	-	-	+	-	-	-	+	3
	-	-	-	+	+	-	-	2
	-	-	-	+	-	+	-	3
	-	-	-	+	-	-	+	1
	-	-	-	-	+	+	-	1
	-	-	-	-	+	-	+	1
	-	-	-	-	-	+	+	2
3-Triple	+	+	-	-	-	+	-	1
	+	+	-	-	-	-	+	1
	+	-	-	-	-	+	+	2
	-	+	+	-	+	-	-	2
	-	+	-	+	-	+	-	1
	-	-	+	-	+	+	-	1
	-	-	+	-	-	+	+	1
	-	-	-	-	+	+	+	2
Total								189

Notes: FluA, Influenza A virus; FluB, Influenza B virus; PIV1, Parainfluenza virus type 1; PIV2, Parainfluenza virus type 2; PIV3, Parainfluenza virus type 3; RSV, Respiratory Syncytial Virus; ADV, Adenovirus. “-”, Negative; “+”, Positive.
